# Targeting the CBP/β-Catenin Interaction to Suppress Activation of Cancer-Promoting Pancreatic Stellate Cells

**DOI:** 10.3390/cancers12061476

**Published:** 2020-06-05

**Authors:** Mingtian Che, Soo-Mi Kweon, Jia-Ling Teo, Yate-Ching Yuan, Laleh G. Melstrom, Richard T. Waldron, Aurelia Lugea, Raul A. Urrutia, Stephen J. Pandol, Keane K. Y. Lai

**Affiliations:** 1 Department of Molecular Medicine, Beckman Research Institute of City of Hope, Duarte, CA 91010, USA; Mingtian.Che@cshs.org (M.C.); skweon@coh.org (S.-M.K.); jteo@coh.org (J.-L.T.); 2 Department of Computational and Quantitative Medicine, Beckman Research Institute of City of Hope, Duarte, CA 91010, USA; YYuan@coh.org; 3 Department of Surgery, City of Hope National Medical Center, Duarte, CA 91010, USA; lmelstrom@coh.org; 4 Pancreatic Research Program, Cedars-Sinai Medical Center, Los Angeles, CA 90048, USA; Richard.Waldron@cshs.org (R.T.W.); Aurelia.Lugea@cshs.org (A.L.); Stephen.Pandol@cshs.org (S.J.P.); 5 Department of Medicine, University of California, Los Angeles (UCLA), Los Angeles, CA 90095, USA; 6 Department of Surgery and the Genomic Sciences and Precision Medicine Center (GSPMC), Medical College of Wisconsin, Milwaukee, WI 53226, USA; rurrutia@mcw.edu; 7 Department of Pathology, City of Hope National Medical Center, and Department of Molecular Medicine, Beckman Research Institute of City of Hope, Duarte, CA 91010, USA; 8 City of Hope Comprehensive Cancer Center, Duarte, CA 91010, USA

**Keywords:** pancreatic cancer, pancreatic stellate cells, Wnt signaling, CBP, p300, pancreatitis, fibrosis

## Abstract

Background: Although cyclic AMP-response element binding protein-binding protein (CBP)/β-catenin signaling is known to promote proliferation and fibrosis in various organ systems, its role in the activation of pancreatic stellate cells (PSCs), the key effector cells of desmoplasia in pancreatic cancer and fibrosis in chronic pancreatitis, is largely unknown. Methods: To investigate the role of the CBP/β-catenin signaling pathway in the activation of PSCs, we have treated mouse and human PSCs with the small molecule specific CBP/β-catenin antagonist ICG-001 and examined the effects of treatment on parameters of activation. Results: We report for the first time that CBP/β-catenin antagonism suppresses activation of PSCs as evidenced by their decreased proliferation, down-regulation of “activation” markers, e.g., α-smooth muscle actin (α-SMA/Acta2), collagen type I alpha 1 (Col1a1), Prolyl 4-hydroxylase, and Survivin, up-regulation of peroxisome proliferator activated receptor gamma (Ppar-γ) which is associated with quiescence, and reduced migration; additionally, CBP/β-catenin antagonism also suppresses PSC-induced migration of cancer cells. Conclusion: CBP/β-catenin antagonism represents a novel therapeutic strategy for suppressing PSC activation and may be effective at countering PSC promotion of pancreatic cancer.

## 1. Introduction

Pancreatic cancer, predominantly comprised of pancreatic ductal adenocarcinoma (PDAC), ranks as the 4th leading cause of cancer deaths in both men and women in the United States, with ~52% of pancreatic cancer patients being diagnosed at an advanced stage of disease for which 5-year survival is a dismal 3% [1]. As such, there is an urgent need for treatments that offer durable benefits to PDAC patients. Treatments for PDAC, which traditionally have focused on targeting pancreatic tumor cells (i.e., parenchymal cells), have been insufficient or have failed for the most part [2,3]. More recently, it has been recognized that activated pancreatic stellate cells (PSCs) (i.e., stromal cells), which are characterized by increased proliferation, up-regulation of “activation” markers, and enhanced migration, promote PDAC progression [2,3,4]. Moreover, the “desmoplasia” of PDAC, i.e., the extensive pro-tumorigenic fibrosis effected by activated PSCs [2,3,4,5], has been found to correlate negatively with patient survival and to be present at similar levels in both primary tumors and metastatic lesions [6]. Thus, activated PSCs represent an attractive therapeutic target to aid in combating PDAC.

The Wnt signaling pathway is an incredibly complex and critical controller of a myriad of processes in mammals, intricately regulating cellular proliferation and cellular differentiation [7,8]. “Canonical” Wnt signaling (or Wnt/β-catenin signaling) is the arm of the pathway associated with β-catenin accumulation in the nucleus and β-catenin forming a complex with members of the TCF/LEF family of transcription factors to regulate target gene transcription. A previous study has shown that retinoic acid-mediated suppression of Wnt/β-catenin signaling suppresses PSC activation in mice with chronic pancreatitis, leading to amelioration of chronic pancreatitis (which itself is a predominant risk factor for PDAC [9,10]) and associated fibrosis [11]. It has been previously demonstrated that, in Wnt/β-catenin signaling, β-catenin recruits either of the Kat3 transcriptional coactivators, cyclic AMP-response element binding protein-binding protein (CBP) or its closely related homolog, p300, to effect transcription and expression of respective target genes [12,13,14,15]. CBP/β-catenin signaling is associated with symmetric non-differentiative proliferation in cancer and fibrosis, whereas p300/β-catenin signaling initiates differentiation and a decrease in cellular potency [12,13,14,15]. Recently, it has been reported that the small molecule specific CBP/β-catenin antagonist ICG-001 [16] suppresses the activation of hepatic stellate cells, which are developmentally and functionally analogous to PSCs [17,18], as well as suppressing associated fibrogenesis in an acute CCl_4_-induced liver injury mouse model [19]. However, the significance of the CBP/β-catenin signaling pathway in PSCs is largely unknown. Thus, the CBP/β-catenin signaling pathway represents a potentially viable, but not yet characterized therapeutic opportunity to target in activated PSCs.

Based on the aforementioned observations, we set out to investigate whether antagonizing the CBP/β-catenin signaling pathway would suppress activation of PSCs and may be useful for combating PDAC and chronic pancreatitis. Herein, we report for the first time that the small molecule specific CBP/β-catenin antagonist ICG-001 suppresses activation of PSCs as evidenced by their decreased proliferation, down-regulation of activation markers, e.g., α-smooth muscle actin (α-SMA/Acta2), collagen type I alpha 1 (Col1a1), Prolyl 4-hydroxylase, and Survivin, up-regulation of peroxisome proliferator activated receptor gamma (Ppar-γ), which is associated with quiescence, and reduced migration; and that migration of PDAC cells is reduced when co-cultured with PSCs which have been pre-treated with ICG-001. Hence, CBP/β-catenin antagonist ICG-001 represents a novel therapeutic option for suppressing PSC activation and promotion of PDAC.

## 2. Results

### 2.1. CBP/β-Catenin Antagonism Suppresses Proliferation of Pancreatic Stellate Cells

**Figure 1:**
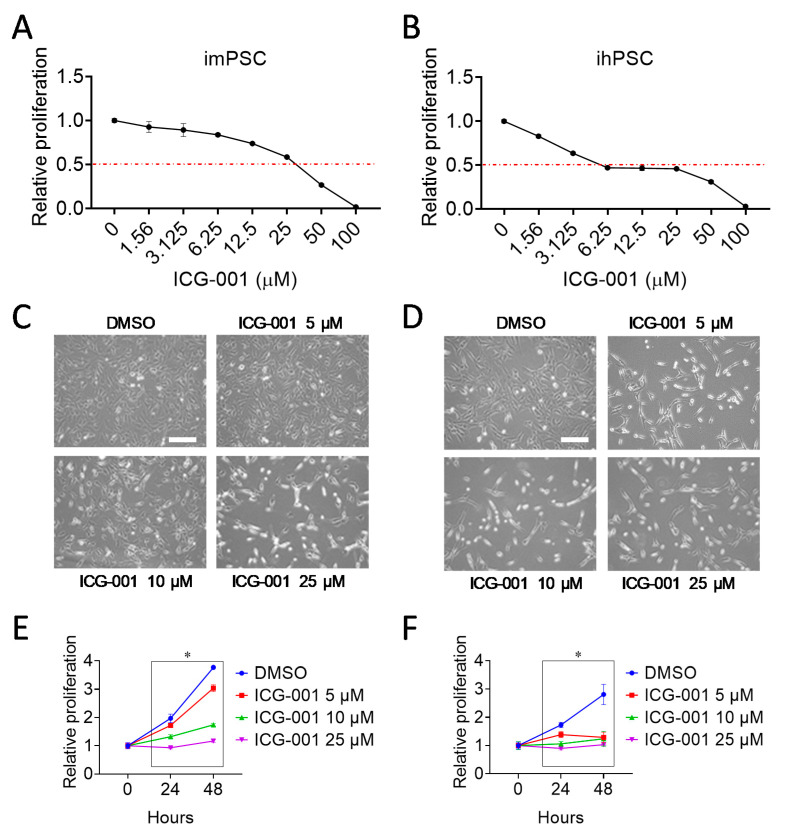
Cyclic AMP-response element binding protein-binding protein (CBP)/β-catenin antagonism suppresses proliferation of pancreatic stellate cells. Effect of CBP/β-catenin antagonist ICG-001 versus control (DMSO) treatment for 48 h on proliferation of immortalized mouse pancreatic stellate cells (imPSC) (**A**) and immortalized human pancreatic stellate cells (ihPSC) (**B**) as assessed by CellTiter-Glo assay. imPSC, immortalized mouse pancreatic stellate cells; ihPSC, immortalized human pancreatic stellate cells. Effect of ICG-001 treatment for 48 h on proliferation of imPSC (**C**) and ihPSC (**D**) as assessed by microscopy. Scale bar: 150 μm. Effect of ICG-001 treatment for 24 h and 48 h on proliferation of imPSC (**E**) and ihPSC (**F**) as assessed by cell counting. *n* = 3, * *p* < 0.05 when each ICG-001 group compared to control (DMSO) at respective time point.

Activated pancreatic stellate cells (PSCs) are known to promote pancreatic ductal adenocarcinoma (PDAC) progression and are characterized by increased proliferation [2,3,4]. To investigate whether inhibition of CBP/β-catenin signaling would suppress proliferation of PSCs, the small molecule specific CBP/β-catenin antagonist ICG-001 [16] versus control (DMSO) was used to treat immortalized mouse PSC line (imPSC) and immortalized human PSC line (ihPSC), which were established as previously described [20,21]. We found that ICG-001 inhibited proliferation of imPSC and ihPSC, as assessed by CellTiter-Glo proliferation assay (Figure 1A,B), microscopy (Figure 1C,D), and cell counting (Figure 1E,F). In addition, ICG-001 IC_50_ of ~25 μM and ranging from ~5 to ~25 uM, for imPSC and ihPSC, respectively, were estimated based on CellTiter-Glo proliferation assay (Figure 1A,B). Furthermore, ICG-001 treatment induces imPSC and ihPSC to change from a more spread out morphology to a thinner or more round, quiescent morphology (Figure 1C,D). Collectively, our results demonstrate that ICG-001 suppresses activation of PSC by inhibiting proliferation and inducing quiescent morphology.

### 2.2. CBP/β-Catenin Antagonism Suppresses Activation Markers of PSCs

**Figure 2:**
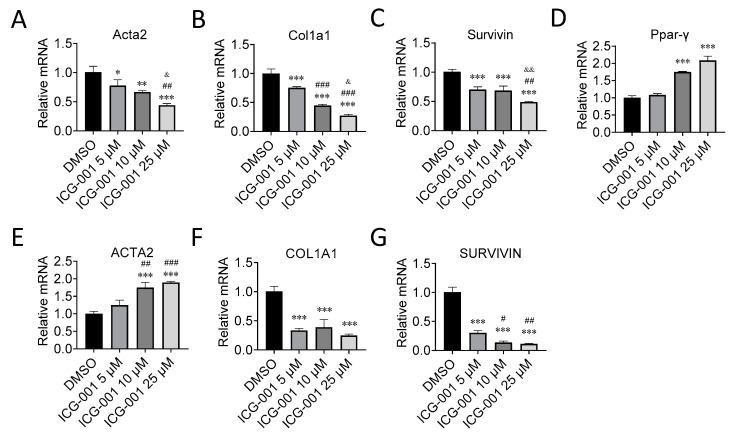
CBP/β-catenin antagonism suppresses gene expression of activation markers of pancreatic stellate cells. Effect of CBP/β-catenin antagonist ICG-001 versus control (DMSO) treatment for 48 h of immortalized mouse pancreatic stellate cells (imPSC) on mRNA expression of activation markers, Acta2 (**A**); Col1a1 (**B**); Survivin (**C**); and Ppar-γ which is associated with quiescence (**D**). Effect of ICG-001 treatment for 48 h of immortalized human pancreatic stellate cells (ihPSC) on mRNA expression of activation markers, ACTA2 (**E**); COL1A1 (**F**); and SURVIVIN (**G**). *n* = 3, * *p* < 0.05, ** *p* < 0.01, *** *p* < 0.001 compared to control (DMSO), # *p* < 0.05, ## *p* < 0.01, ### *p* < 0.001 compared to ICG-001 5 µM, & *p* < 0.05, && *p* < 0.01compared to ICG-001 10 μM.

We next tested whether inhibition of CBP/β-catenin signaling would suppress established activation markers of PSCs, such as *Acta2*, *Col1a1*, and *Survivin (Birc5)* [2,3,4,22,23,24] at the level of mRNA expression. We found that CBP/β-catenin antagonist ICG-001 versus control (DMSO) suppressed in a dose dependent manner, Acta2, Col1a1, and Survivin (Birc5) mRNA expression by up to ~60%, 70%, and 50%, respectively, in imPSC (Figure 2A–C), as assessed by qPCR. Consistent with these findings, we found that ICG-001 also induced mRNA expression of Ppar-γ, which is associated with PSC quiescence [4,22,25,26], up to ~2.1-fold in imPSC. ICG-001 also suppressed *COL1A1* and *SURVIVIN (BIRC5)* mRNA expression by up to ~75% and 90%, respectively, in ihPSC, but interestingly *ACTA2* mRNA expression was induced up to ~1.9-fold, whereas PPAR-γ mRNA expression was not detected (Ct value > 35) (Figure 2E–G).

**Figure 3:**
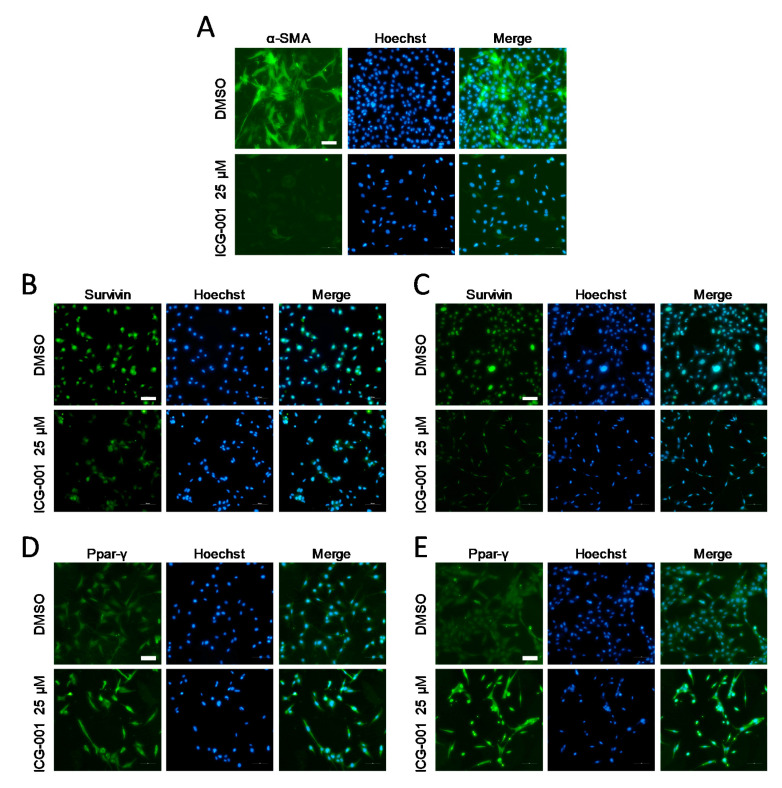
CBP/β-catenin antagonism suppresses protein expression of activation markers of pancreatic stellate cells as assessed by immunofluorescence. Effect of CBP/β-catenin antagonist ICG-001 versus control (DMSO) treatment for 72 h of immortalized mouse pancreatic stellate cells (imPSC) on protein expression of activation markers, Acta2 (α-SMA) (**A**); and Survivin (**B**); and Ppar-γ which is associated with quiescence (**D**); Effect of ICG-001 treatment for 72 h of immortalized human pancreatic stellate cells (ihPSC) on protein expression of activation markers, SURVIVIN (**C**); and PPAR-γ (**E**); Scale bar: 100 µm. Hoechst: Hoechst 33342.

**Figure 4:**
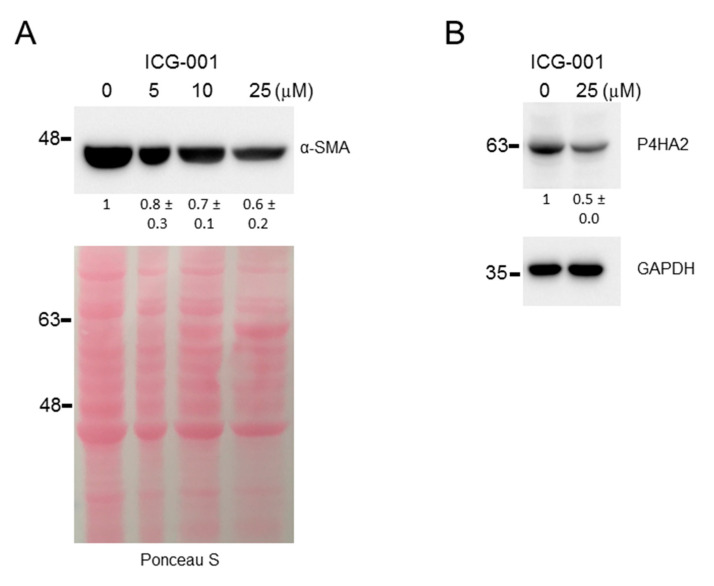
CBP/β-catenin antagonism suppresses protein expression of activation markers of pancreatic stellate cells as assessed by immunoblot. Effect of CBP/β-catenin antagonist ICG-001 versus control (DMSO) treatment for 72 h of immortalized mouse pancreatic stellate cells (imPSC) on protein expression of activation markers, Acta2 (α-SMA) (**A**) and Prolyl 4-hydroxylase (P4HA2) (**B**). Numerical values below protein bands indicate densitometric quantitation normalized to Ponceau S or GAPDH as indicated and then to control (DMSO). Numerical values and associated horizontal marks to the left of protein bands indicate relative position of molecular weight (kDa) markers. (Whole immunoblots are presented in Figure S1.).

We next assessed the effect of ICG-001 on PSC activation and quiescence markers at the level of protein expression, by immunofluorescence or immunoblot. We found that ICG-001 reduced the expression of Acta2 (α-SMA) and Survivin and induced the expression of Ppar-γ in imPSC (Figure 3A,B,D), and similar results were obtained for SURVIVIN and PPAR-γ in ihPSC (Figure 3C,E), as assayed by immunofluorescence. Interestingly, α-SMA was not detected by immunofluorescence in ihPSC, consistent with previous findings, which failed to detect α-SMA at the protein level in this cell line (data not shown) and another immortalized human PSC cell line [27]. Consistent with the immunofluorescence results, ICG-001 reduced the expression of α-SMA by up to ~40% in imPSC (Figure 4A). Given that Prolyl 4-hydroxylase is a central enzyme in the hydroxylation of proline residues in procollagen, serving as a functional indicator of collagen synthesis and thus as another PSC activation marker [26,28], we next tested the effect of ICG-001 on the expression of this marker at the protein level. Immunoblot shows that ICG-001 suppresses Prolyl 4-hydroxylase (P4HA2) by up to ~50% in imPSC (Figure 4B). Thus, based on these protein expression data, along with the aforementioned mRNA expression data, we conclude that CBP/β-catenin antagonism suppresses activation and induces quiescence markers of PSCs.

### 2.3. CBP/β-Catenin Antagonism Suppresses Migration of PSCs and PSC-Induced Migration of Cancer Cells

**Figure 5:**
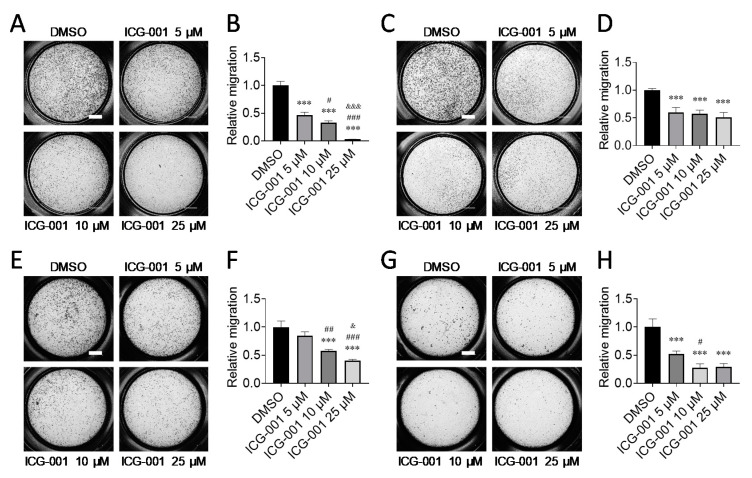
CBP/β-catenin antagonism suppresses migration of pancreatic stellate cells and pancreatic stellate cell-induced migration of pancreatic cancer cells. Effect of CBP/β-catenin antagonist ICG-001 versus control (DMSO) on migration of immortalized mouse pancreatic stellate cells (imPSC) ((**A**), Crystal Violet staining of cells which migrated; and (**B**), relative migration) and on migration of immortalized human pancreatic stellate cells (ihPSC) ((**C**), Crystal Violet staining of cells which migrated; and (**D**), relative migration), as assessed by Transwell migration assay. Note: imPSC and ihPSC were treated with ICG-001 for 48 h, after which time cells were seeded in serum-free medium onto 8-μm Transwell insert, and the lower chamber was filled with 10% FBS medium. Cells were then allowed to migrate for 6 h (imPSC) or 24 h (ihPSC) and kept in corresponding concentrations of ICG-001 versus control (DMSO) during migration. Effect of ICG-001 on imPSC-induced migration of mouse pancreatic cancer cells Panc02 ((**E**), Crystal Violet staining of cells which migrated; and (**F**), relative migration) and on ihPSC-induced migration of human pancreatic cancer cells PANC-1 ((**G**), Crystal Violet staining of cells which migrated; and (**H**), relative migration), as assessed by Transwell migration assay. Note: For PSC-induced Panc02 and PANC-1 cell migration, imPSC and ihPSC were pre-treated with ICG-001 for 72 h, after which time respective PSCs were seeded in 10% FBS medium into the lower chamber and respective cancer cells were seeded in 10% FBS medium onto Transwell insert. Cells were then allowed to migrate for 24 h. Relative migration was determined by counting the number of cells which had migrated across the Transwell insert as assessed by Crystal Violet staining and then normalizing to control (DMSO). *n* = 3, *** *p* < 0.001 compared to control (DMSO), # *p* < 0.05, ## *p* < 0.01, ### *p* < 0.001 compared to ICG-001 5 µM, & *p* < 0.05, &&& *p* < 0.001compared to ICG-001 10 μM. Scale bar: 1 mm.

Next, we tested whether inhibition of CBP/β-catenin signaling would suppress PSC migration which is another established characteristic of activated PSCs [2,3,4]. To do so, we treated imPSC with CBP/β-catenin antagonist ICG-001 versus control (DMSO) and found that ICG-001 treatment suppressed migration by up to ~90%, as assessed by Transwell migration assay (Figure 5A,B). ICG-001 also suppressed migration of ihPSC by up to ~50% (Figure 5C,D). Activated PSCs are known to induce pancreatic cancer cell migration [2,3,4,29,30,31], possibly via PSC-mediated induction of epithelial-mesenchymal transition in cancer cells [31]. Accordingly, we reasoned that pancreatic cancer cells, co-cultured with PSCs, which have been pre-treated with and thus presumably “de-activated” by ICG-001, would exhibit decreased migration compared with pancreatic cancer cells co-cultured with PSCs pre-treated with vehicle control. To test this notion, we co-cultured mouse pancreatic cancer cell line Panc02 with imPSC, which had been pre-treated for 72 h with ICG-001 or control, and assessed Transwell migration of the pancreatic cancer cells. We found that ICG-001 pre-treatment of imPSC, which were subsequently co-cultured with Panc02 cancer cells, suppressed PSC-induced migration of Panc02 cancer cells by up to ~60% (Figure 5E,F). Similarly, we found that ICG-001 pre-treatment of ihPSC, which were subsequently co-cultured with human pancreatic cancer cell line PANC-1, suppressed PSC-induced migration of PANC-1 cancer cells by up to ~70% (Figure 5G,H).

## 3. Discussion

Given that pancreatic cancer, predominantly comprised of pancreatic ductal adenocarcinoma (PDAC), ranks as the 4th leading cause of cancer deaths in the United States with a 5-year survival for advanced stage disease of only 3% [1], there is an urgent need for treatments that offer durable benefits to PDAC patients. With increasing recognition that PDAC treatments traditionally focused on targeting pancreatic tumor cells have been insufficient or failed [2,3] and that activated pancreatic stellate cells (PSCs) promote PDAC progression [2,3,4] and are the key effector cells of desmoplasia [2,3,4,5], which correlates negatively with patient survival [6], there has been an increasing focus on developing novel therapeutic strategies which target activated PSCs to aid in combatting PDAC [2,3,4,5].

We now report for the first time that the small molecule specific CBP/β-catenin antagonist ICG-001 suppresses activation of PSCs as evidenced by their decreased proliferation, down-regulation of activation markers, e.g., Acta2 (in imPSC but apparently not in ihPSC), Col1a1, Prolyl 4-hydroxylase, and Survivin, up-regulation of Ppar-γ which is associated with quiescence, and reduced migration of PSC, as well as by reduced PSC-induced migration of pancreatic cancer cells. Our results are consistent with those of a previous study showing that retinoic acid-mediated suppression of Wnt/β-catenin signaling suppresses PSC activation as evidenced by inhibition of PSC proliferation and Col1a1 expression in vitro and by amelioration of PSC-mediated chronic pancreatitis and associated fibrosis in mice [11]. Our results are also consistent with those of a recent report revealing that ICG-001 suppresses the activation of hepatic stellate cells (which are developmentally and functionally analogous to PSCs) as evidenced by inhibition of α-SMA and collagen-I expression and migration by hepatic stellate cells in vitro, as well as by suppression of associated fibrogenesis in an acute CCl_4_-induced liver injury mouse model [19]. Expression of Acta2, Col1a1, Prolyl 4-hydroxylase, and Survivin is associated with activated PSCs [2,3,4,22,23,24,26,28] which actively proliferate and migrate [4], whereas expression of Ppar-γ is associated with quiescent PSCs [4,22,25,26]. Activated PSCs are the key effector cells for producing the collagen stroma of PDAC, with the resulting fibrous stroma capable of impeding chemotherapeutics/drugs from reaching targets [3]. The interplay between activated PSCs and PDAC cells enhances cancer progression [3], e.g., activated PSCs induce PDAC cell migration which has previously been correlated with epithelial-mesenchymal transition (EMT) [3,31]. Hence, based on our results, we would expect that suppressing activation and inducing quiescence of PSCs by treatment with ICG-001 would have a therapeutically beneficial effect on PSCs and PSC-associated PDAC progression in vivo.

Interestingly, we found in our current study that Acta2 mRNA expression differed between imPSC and ihPSC in response to ICG-001 treatment, with imPSC showing down-regulation and ihPSC showing apparent up-regulation in expression. The exact cause of the observed apparent discrepancy is unknown. A possible explanation for the discrepancy is that imPSC originates from relatively normal tissue whereas ihPSC originates from cancer tissue. Additionally, the Ct value of Acta2 for imPSC treated with control (DMSO) was ~21, whereas the Ct value for ihPSC treated with control (DMSO) was ~27, suggesting that perhaps Acta2 is not substantially expressed at the mRNA level in ihPSC versus imPSC, so that the observed up-regulation in mRNA expression with ICG-001 treatment in ihPSC may not be entirely comparable to the down-regulation in imPSC, given that the baseline Ct values between the two cell lines are so different. Moreover, the immortalization process, together with the difference in tissue of origin, in addition to the inherent biological variability between the different cells, may explain the differential regulation of Acta2 mRNA expression. An analogous explanation could be offered for the observed difference in Ppar-γ mRNA expression between imPSC and ihPSC. Thus and as recently underscored by Lenggenhager et al., cognizance of differences in PSC origin, condition of the pancreas from which PSCs were derived, and whether PSC cultures were primary or immortalized, is important given that such differences may explain apparently contradictory results between experiments using different types of PSCs [27].

In a broader, more fundamental context, Acta2 [32,33], Col1a1 [34], and Survivin [35] have all been previously identified as direct or indirect targets of the CBP/β-catenin signaling pathway and associated with a proliferative and pro-fibrotic phenotype which characterizes activated PSCs [2,3,4,22,23,24]. Furthermore, Ppar-γ, a nuclear receptor which is associated with PSC quiescence [4,22,25,26], is known to have anti-inflammatory/anti-fibrotic activity [36] and should compete with β-catenin for binding to CBP’s N-terminal region, thereby phenocopying ICG-001 antagonism of CBP/β-catenin binding and associated signaling [37]. As such, it is not surprising that various studies have shown that treatment with CBP/β-catenin antagonist ICG-001 (or the structurally related derivative PRI-724) is effective pre-clinically at ameliorating fibrosis in the peritoneum [38], endometrium [39], lung [13], kidney [32], skin [40], heart [41], and liver [19,42]. Importantly, a recent phase 1 trial utilizing PRI-724 demonstrated that treatment of patients safely improved liver histology and Child–Pugh scores for cirrhosis [43], suggesting that CBP/β-catenin antagonism is a viable therapeutic for combating fibrotic diseases in general. Moreover, it is known that chronic pancreatitis is a pathological syndrome characterized by persistent fibrosis effected by activated PSCs and is itself a predominant risk factor for PDAC [9,10], conferring a ~8 to 12-fold increased risk of developing PDAC to chronic pancreatitis patients [44]. These observations, in conjunction with the results of our current study, have overarching therapeutic implications, namely: CBP/β-catenin antagonism would be expected not only to be effective at suppressing activation of PSCs and thereby ameliorating already existing PDAC, but also to be effective as a “PDAC prophylactic” by inhibiting activation of PSCs during the early pre-cancerous stage of pancreatic fibrosis/chronic pancreatitis.

Given the pressing need to develop and implement better treatment strategies for combatting PDAC, we now present in this Communication our novel results on the effectiveness of CBP/β-catenin antagonism in suppressing PSC activation, with broad therapeutic implications for treating PDAC and chronic pancreatitis, both of which are known to be promoted by activated PSCs. Because of the limitation in scope of our current study, future studies (e.g., using primary PSCs and in vivo models) would be required to further validate the effect of CBP/β-catenin antagonism on PSC biology/pathobiology, including the interaction between PSCs and PDAC cells.

## 4. Materials and Methods

### 4.1. Cell Lines and Culture Conditions

Immortalized mouse pancreatic stellate cell line (imPSC) and immortalized human pancreatic stellate cell line (ihPSC) were kindly provided by Richard T. Waldron, Aurelia Lugea, and Raul A. Urrutia and were established as previously described [20,21]. imPSC were grown and cultivated in Dulbecco’s Modified Eagle Medium (DMEM) with low glucose (1000 mg/L) while ihPSC were grown and cultivated in DMEM with high glucose (4500 mg/L). All culture medium was supplemented with 10% fetal bovine serum (FBS) and 1% penicillin-streptomycin unless otherwise indicated. Cells were maintained in an incubator at 37 °C with 5% CO_2_.

### 4.2. Pharmacologic Agents

Small molecule specific CBP/β-catenin antagonist ICG-001 as previously described [16] was donated by Professor Michael Kahn and used at concentrations as indicated.

### 4.3. Cell Proliferation Assays

CellTiter-Glo assay (Promega) was performed according to the manufacturer’s protocol. Cells were plated in triplicate in 96-well plates at 1 × 10^4^ cells/well in 100 μL of medium. Plates were incubated at 37 °C in 5% CO_2._ The next day, cells were treated with ICG-001 at 100 μM, 50 μM, 25 μM, 12.5 μM, 6.25 μM, 3.13 μM 1.56 μM or control (DMSO) and were incubated for an additional 48 h. Then, 50 µL CellTiter-Glo reagent and 50 µL of DMEM were added to the wells and incubated for 10 min protected from the light. Luminescence signal was assayed using EnVision Multilabel Plate Reader (Perkin-Elmer).

Cell proliferation was also assessed by cell counting using a hemocytometer. Briefly, imPSC or ihPSC were seeded in 6-well plates at 5 × 10^4^ cells/well and incubated at 37 °C in 5% CO_2._ The next day, cells were treated with ICG-001 at 5 μM, 10 μM, 25 μM or control (DMSO) for 24 h or 48 h. Cell numbers were counted at 0, 24, and 48 h after treatment.

### 4.4. Quantitative Polymerase Chain Reaction (qPCR)

Cells were treated with ICG-001 for 48 h. Total mRNA was extracted by TRIzol reagent (Invitrogen) according to the manufacturer’s protocol. cDNA was synthesized using qScript cDNA Synthesis Kit (Quantabio) and used as template for qPCR with SYBR Green detection method. The PCR primer sequences used for mouse cells were as follows: Acta2 (F: 5′-GTCCCAGACATCAGGGAGTAA-3′, R: 5′-TCGGATACTTCAGCGTCAGGA-3′); Col1a1 (F: 5′-GCTCCTCTTAGGGGCCACT-3′, R: 5′-CCACGTCTCACCATTGGGG-3′); Survivin (F: 5′-GAGGCTGGCTTCATCCACTG, R: 5′-ATGCTCCTCTATCGGGTTGTC-3′); Ppar-γ (F: 5′-TTTTCCGAAGAACCATCCGATT-3′, R: 5′-ATGGCATTGTGAGACATCCCC-3′). The PCR primer sequences used for human cells were as follows: ACTA2 (F: 5′-CTATGAGGGCTATGCCTTGCC-3′, R: 5′-GCTCAGCAGTAGTAACGAAGGA-3′); COL1A1 (F: 5′-GAGGGCCAAGACGAAGACATC-3′, R: 5′-CAGATCACGTCATCGCACAAC-3′); SURVIVIN (F: 5′-AGGACCACCGCATCTCTACAT-3′, R: 5′-AAGTCTGGCTCGTTCTCAGTG-3′); PPAR-γ (F: 5′-CTATGGAGTTCATGCTTGTG-3′, R: 5′-GTACTGACATTTATTT-3′). Housekeeping gene PCR primer sequences used were GAPDH for mouse cells (F: 5′-GGTGCTGAGTATGTCGTGGA-3′, R: 5′-ACAGTCTTCTGGGTGGCAGT-3′) and GAPDH for human cells (F: 5′-AGAAGGCTGGGGCTCATTTG-3′, R: 5′ AGGGGCCATCCACAGTCTTC-3′).

### 4.5. Immunofluorescence

Cells were plated, and the next day cells were treated with ICG-001 or control (DMSO) for 72 h, followed by 4% PFA fixation for 10 min. After 3 times of PBS washing, 1% BSA with 0.1% Triton X-100 was used for blocking nonspecific binding. Primary antibodies for α-SMA (Cell Signaling Technology, #19245s; 1:100), Survivin (Cell Signaling Technology, #2808s; 1:250), and Ppar-γ (Affinity BioReagents, #PA3-821; 1:50) were used for overnight incubation at 4 °C. Secondary antibody anti-rabbit IgG-Alexa Fluor 488 (Invitrogen, #A11034; 1:1000) was incubated for 40 min at room temperature. Hoechst 33342 was used for nuclear staining for 10 min. A fluorescence microscope (Eclipse Ti2, Nikon) was used to observe target protein expression.

### 4.6. Western Blot

2 × 10^5^ of imPSC were plated in 10 cm plates. The next day, cells were treated with ICG-001 or control (DMSO) and were incubated for an additional 72 h. Then, cells were collected, and total cellular proteins were extracted using M-PER Mammalian Protein Extraction Reagent (ThermoFisher). After protein quantification by Bradford assay method, protein samples were separated by 10% SDS PAGE, followed by transfer to nitrocellulose membrane. Next, the membranes were blocked with 5% milk in Tris-Buffered Saline with 0.1% Tween. The membranes were incubated with primary antibody overnight at 4 °C and incubated with secondary antibody for 1 h the following day. Primary antibodies for α-SMA (Cell Signaling Technology, #19245s), Prolyl 4-hydroxylase (P4HA2) (ThermoFisher, #PA5-96280), and GAPDH (Santa Cruz Biotechnologies, #sc-32233), and secondary antibody anti-rabbit IgG-HRP (Santa Cruz Biotechnologies, #sc-2357) were used. Protein bands were detected using ECL prime Western blotting detection reagent (Amersham) and visualized by Chemidoc Imaging System (Bio-Rad). Each protein band of interest was digitized by densitometry program ImageJ (NIH) or ImageLab (Bio-Rad). Densitometric quantitation of protein bands was normalized to Ponceau S or GAPDH and then to control (DMSO).

### 4.7. Transwell Migration Assay

For PSC Transwell migration assay, imPSC and ihPSC were treated with ICG-001 for 48 h. Then, 1 × 10^4^ cells were seeded in serum-free DMEM onto 8-μm Transwell insert (Corning). The lower chamber was filled with 10% FBS supplemented DMEM. Cells were then allowed to migrate while incubated at 37 °C with 5% CO_2_ for 6 h (imPSC) or 24 h (ihPSC) and kept in corresponding concentrations of ICG-001 versus control (DMSO) during migration.

For PSC-induced Panc02 and PANC-1 cancer cell Transwell migration assay, imPSC and ihPSC were pre-treated with ICG-001 for 72 h, and then seeded in 10% FBS DMEM into the lower chamber of 24-well plate at a total number of 5 × 10^4^ (imPSC) or 1 × 10^5^ (ihPSC) cells per well. Panc02 or PANC-1 cells (1 × 10^4^) were seeded in 10% FBS DMEM onto Transwell insert. Cells were then incubated and allowed to migrate for 24 h.

For both PSC Transwell migration assay and PSC-induced Panc02 and PANC-1 cancer cell Transwell migration assay, after incubation, cells were stained with 1% Crystal Violet solution (Sigma) for 30 min. The cells on the upper surface of the Transwell insert were gently removed by cotton swab, and the cells which had migrated to the bottom surface of the insert were counted under bright field microscopy. Relative migration was determined by normalizing the number of cells which had migrated with ICG-001 treatment to the number of cells which had migrated with control (DMSO) treatment.

### 4.8. Statistical Analysis

Numerical data were expressed as the means ± SD unless otherwise noted. Student’s t-test was performed to assess the statistical significance between two sets of data as appropriate. One-way ANOVA followed by post-hoc Tukey test was performed for multiple comparisons when appropriate. *p* values less than 0.05 were considered significant.

## 5. Conclusions

We report for the first time that the small molecule specific CBP/β-°C antagonist ICG-001 suppresses activation of PSCs as evidenced by their decreased proliferation, down-regulation of activation markers, e.g., Acta2, Col1a1, Prolyl 4-hydroxylase, and Survivin, up-regulation of Ppar-γ which is associated with quiescence, and reduced migration; furthermore, migration of PDAC cells is reduced when co-cultured with PSCs which have been pre-treated with ICG-001. Hence, CBP/β-catenin antagonism represents a novel therapeutic strategy for suppressing PSC activation and may be effective at treating PDAC and “pre-cancerous” chronic pancreatitis, both of which are known to be promoted by activated PSCs.

## Supplementary Materials

The following are available online at https://www.mdpi.com/2072-6694/12/6/1476/s1, Figure S1: CBP/β-catenin antagonism suppresses protein expression of activation markers of pancreatic stellate cells as assessed by immunoblot (Whole immunoblots).

## References

[1] American Cancer Society (2019). Cancer Facts & Figures 2019.

[2] Apte M.V., Wilson J.S., Lugea A., Pandol S.J. (2013). A starring role for stellate cells in the pancreatic cancer microenvironment. Gastroenterology.

[3] Pothula S.P., Pirola R.C., Wilson J.S., Apte M.V. (2020). Pancreatic stellate cells: Aiding and abetting pancreatic cancer progression. Pancreatology.

[4] Xue R., Jia K., Wang J., Yang L., Wang Y., Gao L., Hao J. (2018). A rising star in pancreatic diseases: Pancreatic stellate cells. Front Physiol..

[5] Apte M.V., Park S., Phillips P.A., Santucci N., Goldstein D., Kumar R.K., Ramm G.A., Buchler M., Friess H., McCarroll J.A. (2004). Desmoplastic reaction in pancreatic cancer: Role of pancreatic stellate cells. Pancreas.

[6] Whatcott C.J., Diep C.H., Jiang P., Watanabe A., LoBello J., Sima C., Hostetter G., Shepard H.M., Von Hoff D.D., Han H. (2015). Desmoplasia in primary tumors and metastatic lesions of pancreatic cancer. Clin. Cancer Res..

[7] Clevers H., Nusse R. (2012). Wnt/β-catenin signaling and disease. Cell.

[8] Monga S.P. (2015). β-Catenin Signaling and Roles in Liver Homeostasis, Injury, and Tumorigenesis. Gastroenterology.

[9] Lew D., Afghani E., Pandol S. (2017). Chronic pancreatitis: Current status and challenges for prevention and treatment. Dig. Dis. Sci..

[10] Whitcomb D.C., Shelton C.A., Brand R.E. (2015). Genetics and genetic testing in pancreatic cancer. Gastroenterology.

[11] Xiao W., Jiang W., Shen J., Yin G., Fan Y., Wu D., Qiu L., Yu G., Xing M., Hu G. (2015). Retinoic acid ameliorates pancreatic fibrosis and inhibits the activation of pancreatic stellate cells in mice with experimental chronic pancreatitis via suppressing the Wnt/β-catenin signaling pathway. PLoS ONE.

[12] Teo J.L., Ma H., Nguyen C., Lam C., Kahn M. (2005). Specific inhibition of CBP/beta-catenin interaction rescues defects in neuronal differentiation caused by a presenilin-1 mutation. Proc. Natl. Acad. Sci. USA.

[13] Henderson W.R., Chi E.Y., Ye X., Nguyen C., Tien Y.T., Zhou B., Borok Z., Knight D.A., Kahn M. (2010). Inhibition of Wnt/beta-catenin/CREB binding protein (CBP) signaling reverses pulmonary fibrosis. Proc. Natl. Acad. Sci. USA.

[14] Lai K.K.Y., Nguyen C., Lee K.S., Lee A., Lin D.P., Teo J.L., Kahn M. (2019). Convergence of canonical and Non-canonical Wnt signal: Differential Kat3 coactivator usage. Curr. Mol. Pharmacol..

[15] Thomas P.D., Kahn M. (2016). Kat3 coactivators in somatic stem cells and cancer stem cells: Biological roles, evolution, and pharmacologic manipulation. Cell Biol. Toxicol..

[16] Emami K.H., Nguyen C., Ma H., Kim D.H., Jeong K.W., Eguchi M., Moon R.T., Teo J.L., Oh S.W., Kim H.Y. (2004). A small molecule inhibitor of beta-catenin/CREB-binding protein transcription [corrected]. Proc. Natl. Acad. Sci. USA.

[17] Omary M.B., Lugea A., Lowe A.W., Pandol S.J. (2007). The pancreatic stellate cell: A star on the rise in pancreatic diseases. J. Clin. Investig..

[18] Buchholz M., Kestler H.A., Holzmann K., Ellenrieder V., Schneiderhan W., Siech M., Adler G., Bachem M.G., Gress T.M. (2005). Transcriptome analysis of human hepatic and pancreatic stellate cells: Organ-specific variations of a common transcriptional phenotype. J. Mol. Med. (Berl).

[19] Akcora B., Storm G., Bansal R. (2018). Inhibition of canonical WNT signaling pathway by β-catenin/CBP inhibitor ICG-001 ameliorates liver fibrosis in vivo through suppression of stromal CXCL12. Biochim. Biophys. Acta Mol. Basis Dis..

[20] Cao Y., Szabolcs A., Dutta S.K., Yaqoob U., Jagavelu K., Wang L., Leof E.B., Urrutia R.A., Shah V.H., Mukhopadhyay D. (2010). Neuropilin-1 mediates divergent R-Smad signaling and the myofibroblast phenotype. J. Biol. Chem..

[21] Mathison A., Liebl A., Bharucha J., Mukhopadhyay D., Lomberk G., Shah V., Urrutia R. (2010). Pancreatic stellate cell models for transcriptional studies of desmoplasia-associated genes. Pancreatology.

[22] Apte M.V., Pirola R.C., Wilson J.S. (2012). Pancreatic stellate cells: A starring role in normal and diseased pancreas. Front Physiol..

[23] De Minicis S., Seki E., Uchinami H., Kluwe J., Zhang Y., Brenner D.A., Schwabe R.F. (2007). Gene expression profiles during hepatic stellate cell activation in culture and in vivo. Gastroenterology.

[24] Mantoni T.S., Schendel R.R., Rödel F., Niedobitek G., Al-Assar O., Masamune A., Brunner T.B. (2008). Stromal SPARC expression and patient survival after chemoradiation for non-resectable pancreatic adenocarcinoma. Cancer Biol. Ther..

[25] Jaster R., Lichte P., Fitzner B., Brock P., Glass A., Karopka T., Gierl L., Koczan D., Thiesen H.J., Sparmann G. (2005). Peroxisome proliferator-activated receptor gamma overexpression inhibits pro-fibrogenic activities of immortalised rat pancreatic stellate cells. J. Cell Mol. Med..

[26] Masamune A., Kikuta K., Satoh M., Sakai Y., Satoh A., Shimosegawa T. (2002). Ligands of peroxisome proliferator-activated receptor-gamma block activation of pancreatic stellate cells. J. Biol. Chem..

[27] Lenggenhager D., Amrutkar M., Sántha P., Aasrum M., Löhr J.M., Gladhaug I.P., Verbeke C.S. (2019). Commonly used pancreatic stellate cell cultures differ phenotypically and in their interactions with pancreatic cancer cells. Cells.

[28] Masamune A., Satoh M., Kikuta K., Suzuki N., Shimosegawa T. (2003). Establishment and characterization of a rat pancreatic stellate cell line by spontaneous immortalization. World J. Gastroenterol..

[29] Hwang R.F., Moore T., Arumugam T., Ramachandran V., Amos K.D., Rivera A., Ji B., Evans D.B., Logsdon C.D. (2008). Cancer-associated stromal fibroblasts promote pancreatic tumor progression. Cancer Res..

[30] Vonlaufen A., Joshi S., Qu C., Phillips P.A., Xu Z., Parker N.R., Toi C.S., Pirola R.C., Wilson J.S., Goldstein D. (2008). Pancreatic stellate cells: Partners in crime with pancreatic cancer cells. Cancer Res..

[31] Kikuta K., Masamune A., Watanabe T., Ariga H., Itoh H., Hamada S., Satoh K., Egawa S., Unno M., Shimosegawa T. (2010). Pancreatic stellate cells promote epithelial-mesenchymal transition in pancreatic cancer cells. Biochem. Biophys. Res. Commun..

[32] Hao S., He W., Li Y., Ding H., Hou Y., Nie J., Hou F.F., Kahn M., Liu Y. (2011). Targeted inhibition of β-catenin/CBP signaling ameliorates renal interstitial fibrosis. J. Am. Soc. Nephrol..

[33] Zhou B., Liu Y., Kahn M., Ann D.K., Han A., Wang H., Nguyen C., Flodby P., Zhong Q., Krishnaveni M.S. (2012). Interactions between β-catenin and transforming growth factor-β signaling pathways mediate epithelial-mesenchymal transition and are dependent on the transcriptional co-activator cAMP-response element-binding protein (CREB)-binding protein (CBP). J. Biol. Chem..

[34] Rong M., Chen S., Zambrano R., Duncan M.R., Grotendorst G., Wu S. (2016). Inhibition of β-catenin signaling protects against CTGF-induced alveolar and vascular pathology in neonatal mouse lung. Pediatr. Res..

[35] Ma H., Nguyen C., Lee K.S., Kahn M. (2005). Differential roles for the coactivators CBP and p300 on TCF/beta-catenin-mediated survivin gene expression. Oncogene.

[36] Belvisi M.G., Hele D.J., Birrell M.A. (2006). Peroxisome proliferator-activated receptor gamma agonists as therapy for chronic airway inflammation. Eur. J. Pharmacol..

[37] Ono M., Lai K.K.Y., Wu K., Nguyen C., Lin D.P., Murali R., Kahn M. (2018). Nuclear receptor/Wnt beta-catenin interactions are regulated via differential CBP/p300 coactivator usage. PLoS ONE.

[38] Ji S., Deng H., Jin W., Yan P., Wang R., Pang L., Zhou J., Zhang J., Chen X., Zhao X. (2017). Beta-catenin participates in dialysate-induced peritoneal fibrosis. FEBS Open Bio..

[39] Hirakawa T., Nasu K., Miyabe S., Kouji H., Katoh A., Uemura N., Narahara H. (2019). β-catenin signaling inhibitors ICG-001 and C-82 improve fibrosis in preclinical models of endometriosis. Sci. Rep..

[40] Beyer C., Reichert H., Akan H., Mallano T., Schramm A., Dees C., Palumbo-Zerr K., Lin N.Y., Distler A., Gelse K. (2013). Blockade of canonical Wnt signalling ameliorates experimental dermal fibrosis. Ann. Rheum. Dis..

[41] Blyszczuk P., Müller-Edenborn B., Valenta T., Osto E., Stellato M., Behnke S., Glatz K., Basler K., Lüscher T.F., Distler O. (2017). Transforming growth factor-β-dependent Wnt secretion controls myofibroblast formation and myocardial fibrosis progression in experimental autoimmune myocarditis. Eur. Heart J..

[42] Tokunaga Y., Osawa Y., Ohtsuki T., Hayashi Y., Yamaji K., Yamane D., Hara M., Munekata K., Tsukiyama-Kohara K., Hishima T. (2017). Selective inhibitor of Wnt/β-catenin/CBP signaling ameliorates hepatitis C virus-induced liver fibrosis in mouse model. Sci. Rep..

[43] Kimura K., Ikoma A., Shibakawa M., Shimoda S., Harada K., Saio M., Imamura J., Osawa Y., Kimura M., Nishikawa K. (2017). Safety, Tolerability, and preliminary efficacy of the anti-fibrotic small molecule PRI-724, a CBP/β-catenin inhibitor, in patients with hepatitis C virus-related cirrhosis: A single-center, open-label, dose escalation phase 1 trial. EBioMedicine.

[44] Kirkegård J., Mortensen F.V., Cronin-Fenton D. (2017). Chronic pancreatitis and pancreatic cancer risk: A systematic review and meta-analysis. Am. J. Gastroenterol..

