# Supplementary material for: Targeting the CBP/β-Catenin Interaction to Suppress Activation of Cancer-Promoting Pancreatic Stellate Cells
Source: Cancers (Basel). 2020 Jun 5;12(6):1476. doi: 10.3390/cancers12061476 (PMC7352534; doi:10.3390/cancers12061476)
Supplement: Supplementary file 1 [file cancers-12-01476-s001.pdf]

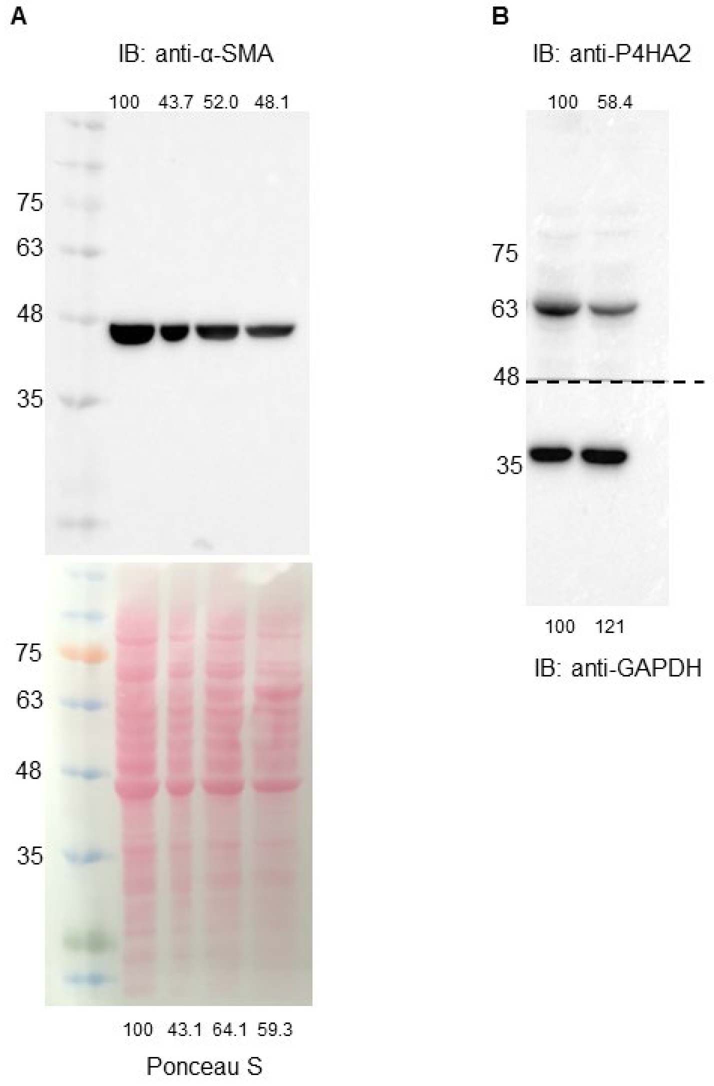

Figure S1. CBP/β-catenin antagonism suppresses protein expression of activation markers of pancreatic stellate cells as assessed by immunoblot. Whole immunoblots corresponding to the immunoblots presented in Figure 4 of the manuscript proper. Effect of CBP/β-catenin antagonist ICG-001 versus control (DMSO) treatment for 72 h of immortalized mouse pancreatic stellate cells (imPSC) on protein expression of activation markers, Acta2 (α-SMA) (A) and Prolyl 4-hydroxylase (P4HA2) (B). Numerical values above or below blots indicate densitometric quantitation normalized to respective control (DMSO). Numerical values to the left of blots indicate relative position of molecular weight (kDa) markers. Dashed line indicates where blot was cut.
